# Efficacy of using trimethoprim‐sulfamethoxazole prophylaxis in an adult patient with autoimmune neutropenia

**DOI:** 10.1002/jgf2.254

**Published:** 2019-05-13

**Authors:** Naoto Azuma, Masahiro Sekiguchi, Hitomi Matsunaga, Hajime Sano, Kiyoshi Matsui

**Affiliations:** ^1^ Division of Rheumatology, Department of Internal Medicine Hyogo College of Medicine Nishinomiya Japan; ^2^ Department of Internal Medicine Hyogo Prefectural Nishinomiya Hospital Nishinomiya Japan; ^3^ Kyoto Okamoto Memorial Hospital Kyoto Japan

**Keywords:** adult, autoimmune neutropenia, prophylaxis, trimethoprim‐sulfamethoxazole

## Abstract

A 63‐year‐old man was admitted for pneumonia with neutropenia. After the pneumonia resolved with administration of antibiotics and granulocyte colony‐stimulating factor, he was diagnosed with autoimmune neutropenia (AIN) on the basis of bone marrow findings and positive antineutrophil antibodies. He had recurring high fever once or twice a month with productive cough and an elevated serum C‐reactive protein level. However, after the initiation of treatment with oral trimethoprim‐sulfamethoxazole (TMP/SMX), the respiratory tract infection no longer recurred. The standard management for adults with AIN has not been established. In our case, the recurrent infection was resolved with TMP/SMX prophylaxis. Thus, TMP/SMX prophylaxis may be beneficial for managing adults with AIN.

## 1 INTRODUCTION

A high incidence of infections has been observed in patients with autoimmune neutropenia (AIN). However, the standard management for patients with AIN has not been established^1^. Here, we report the first case of an adult patient with AIN‐associated recurrent fever and respiratory tract infection that were successfully managed with trimethoprim‐sulfamethoxazole (TMP/SMX) prophylaxis.

## 2 CASE REPORT

**Figure 1:**
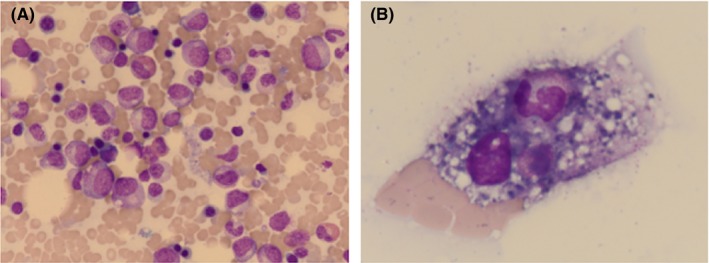
Bone marrow smears revealed hypercellularity with a reduced number of segmented neutrophils without malignant cells and dysplasia (A), and phagocytosis of granulocytes by macrophages (B)

A 63‐year‐old Japanese man with immune thrombocytopenic purpura (ITP) and diabetes mellitus was admitted because of acute pneumonia along with neutropenia. Upon admission, he was not under treatment with corticosteroids or immunosuppressants for ITP. His pneumonia improved with antibiotic therapy (antibiotics used unknown) and the administration of granulocyte colony‐stimulating factor (G‐CSF). The patient had reported a similar episode 2 years ago. After his pneumonia had resolved, he was hospitalized for further examinations related to neutropenia. Hematological examination revealed neutropenia (white blood cell count = 1850/µL: metamyelocytes, 1.0%; stab neutrophils, 6.0%; segmented neutrophils, 3.0%; lymphocytes, 53.0%; monocytes, 20.0%; eosinophils, 15.0%; and basophils, 2.0%) and thrombocytopenia (3.5 × 10^4^/µL). Bone marrow analysis (Figure 1) showed hypercellularity with a reduced number of mature neutrophils and evidence of arrested maturation (nucleated cell count, 34.8 × 10^4^/µL; megakaryocyte count, 167/µL; myeloid/erythroid ratio, 1.9; myeloblasts, 1.6%; promyelocytes, 3.9%; myelocytes, 13.1%; metamyelocytes, 12.9%; stab neutrophils, 17.8%; and segmented neutrophils, 3.3%). Phagocytosis of granulocytes by macrophages was observed. No malignant cells or dysplasia was noted, and the karyotype was normal. The results of the liver and renal function tests were all normal, as were serum albumin, vitamin B_12_, and folic acid levels. C‐reactive protein (CRP) was weakly positive (0.8 mg/dL). The results of the serological tests for hepatitis B virus, hepatitis C virus, human immunodeficiency virus, cytomegalovirus, and human parvovirus B19 were negative, and antibodies to Epstein‐Barr virus indicated a previous infection pattern. The serum β‐D‐glucan level was within the normal range. A 13C‐urea breath test did not detect *Helicobacter pylori*. Autoimmune antibodies, including rheumatoid factor, anticyclic citrullinated peptide antibody, antinuclear antibody, anti–double‐stranded DNA antibody, anti‐Sm antibody, anti‐RNP antibody, anti–SS‐A antibody, myeloperoxidase‐antineutrophil cytoplasmic antibody (ANCA), and proteinase‐3‐ANCA, were negative. A granulocyte immunofluorescence test showed that the patient was positive for antineutrophil antibodies (human neutrophil antigen [HNA]‐1a, HNA‐1b, HNA‐2, HNA‐3a, HNA‐4a, and 9a). Elevated levels of serum G‐CSF (enzyme‐linked immunosorbent assays [ELISA]: 66.7 [normal: <39] pg/mL) and platelet‐associated immunoglobulin (ELISA: 95 [normal: <46] ng/10^7^ cells) were observed. No symptoms and physical findings suggestive of infections and connective tissue diseases were found. His usual medication, glimepiride, was initiated later than the onset of neutropenia and was not changed. No other causes of neutropenia, such as drug‐induced conditions, infections, or connective tissue diseases, could be identified. On the basis of the bone marrow findings and positive antineutrophil antibodies, the patient was diagnosed as having secondary AIN coexisting with ITP.

**Figure 2:**
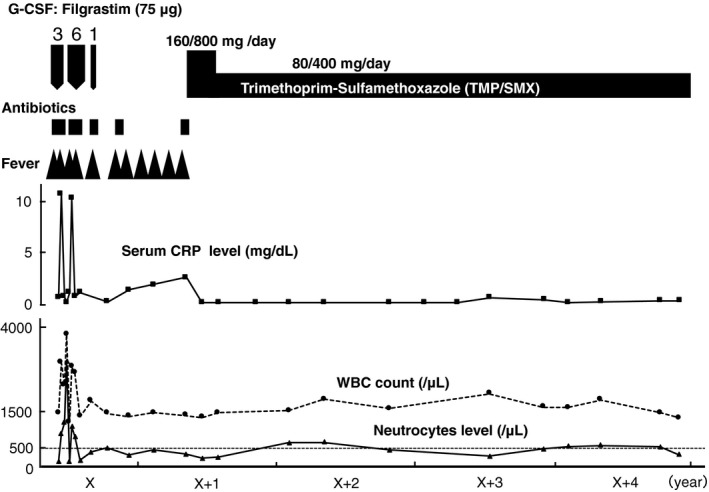
Clinical course; G‐CSF, granulocyte colony‐stimulating factor (subcutaneous injection); 3, for 3 consecutive days; 6, for 6 consecutive days; and 1, for only 1 d. CRP, C‐reactive protein; WBC, white blood cell

From approximately 5 months after his pneumonia ameliorated, he began to have recurring high fever once or twice monthly, with productive cough and elevated serum CRP level (maximum: 10.9 mg/dL). Gastrointestinal symptoms were not observed. Urinalysis revealed no infectious agents. Apparent computed tomographic findings suggestive of infection in his chest, abdomen, and pelvis were not observed. No pathogenic microorganisms were identified. According to the patient, he often had fever accompanied by productive cough. After every administration of antibiotic (ceftriaxone, cefpodoxime, levofloxacin, or moxifloxacin) and G‐CSF, his symptoms improved and serum CRP level decreased promptly. The amelioration and recurrence of symptoms continued for approximately 1 year. However, after the initiation of oral TMP/SMX (160/800 mg/d) (body weight: 82 kg), his fever did not recur, and the CRP level was normalized. TMP/SMX was continuously administered. Two months after the initiation of treatment, we changed the dose of TMP/SMX to 80/400 mg/d. Subsequently, although his neutropenia persisted, no signs of recurrence, including respiratory tract infection, were observed for more than 3 years (Figure 2). Moreover, no TMP/SMX‐induced adverse effects were observed.

## 3 DISCUSSION

Autoimmune neutropenia is a rare disorder characterized by neutropenia and the presence of antineutrophil antibodies. Neutropenia is caused by a peripheral destruction of neutrophils through agglutination or phagocytosis by antibodies against human neutrophil antigens^2^. Primary AIN occurs predominantly in infants and children, whereas secondary AIN is more commonly observed in adults and is associated with underlying autoimmune diseases, malignancies, infections, or drug exposure^2^. The clinical manifestation of AIN, whether primary or secondary, is mainly recurrent infections.

Bux et al^3^ reported that 80% of infants with AIN suffered from mild infections, particularly upper respiratory infections, otitis media, and skin infections, at the time of diagnosis; 12% of patients had severe infections, such as pneumonia, meningitis, or sepsis. Because the clinical course of AIN in infants is generally uncomplicated, not accompanied by severe infections, and resolves spontaneously within months to years, specific treatment for neutropenia and antibiotic prophylaxis are not required in most patients^3^, ^4^. Despite the expected recovery from neutropenia, patients with AIN appear to have a higher incidence of infections during the neutropenic period^4^. Several treatments have been proposed to increase the absolute neutrophil count. The most effective treatment for AIN is the administration of G‐CSF^1^, ^3^, and the effect was observed within a few days in almost all patients^3^. Treatment with G‐CSF is considered beneficial for patients with severe infections or before surgical intervention^3^. Corticosteroids and intravenous immunoglobulin (IVIG) have limited effects^1^, ^3^. In addition, corticosteroids affect host defense, and its long‐term use can induce various side effects. IVIG has only a transient effect, and it is expensive. Previous reports suggested that the prophylactic use of antibiotics, such as cotrimoxazole^3^ and TMP/SMX^4^, in infants with AIN who present with recurrent infections is beneficial. Kobayashi et al^4^ demonstrated that the incidence of infectious complications in infants with AIN accompanied by the prophylactic use of TMP/SMX was significantly less than that in the infants who did not receive TMP/SMX prophylaxis. In adults, spontaneous resolution of AIN is uncommon^2^. Moreover, aging and diabetes can exacerbate a compromised health status, as indicated herein. Therefore, we considered that the importance of antibiotic prophylaxis administration in adults with AIN differed from that of infants.

The organisms responsible for the infections in patients with AIN are not usually identified^4^. Although the pathogenic microorganisms were not identified even in this case, we supposed that the patient with AIN developed bacterial respiratory tract infection repeatedly, on the basis of his symptoms and the effectiveness of antibiotic therapy and G‐CSF administration. We administered TMP/SMX to a patient based on the report on infants, as shown by Kobayashi et al^4^ TMP/SMX has a broad spectrum of activity against bacteria. The antibiotic is thought to have utility in the treatment and prevention of bacterial infections owing to its antimicrobial efficacy, high tissue penetration, and low cost^4^, ^5^, ^6^. On the basis of the guidelines for the management of febrile neutropenia (FN) after chemotherapy for malignancy^7^, ^8^, fluoroquinolone prophylaxis should be considered for high‐risk patients with expected prolonged and profound neutropenia (absolute neutrophil count of <100 cells/mm^3^ for >7 days). A systematic review on antibiotic prophylaxis in patients with neutropenia induced by chemotherapy or following bone marrow transplantation demonstrated that TMP/SMX as well as quinolones reduced the risks of infection‐related mortality, febrile episodes, clinically documented infections, microbiologically documented infections, gram‐negative infections, gram‐positive infections, and bacteremia as compared to no intervention.^9^ To the best of our knowledge, no reports addressing the efficacy of antibiotic prophylaxis in adults with AIN are available. In the reference guide for adult chronic neutropenia,^10^ no findings were reported on the kind of antibiotic recommended for antibiotic prophylaxis in adult patients with chronic neutropenia, including AIN. However, whether TMP/SMX is most suitable for this purpose is still unknown^1^; nevertheless, the present clinical course clearly demonstrated the efficacy of using TMP/SMX prophylaxis. Kobayashi et al^4^ administered 8/40 mg TMP/SMX per kg body weight per day orally to infants until they recovered from neutropenia. In our case, a lower dose of TMP/SMX demonstrated a prophylactic effect. Although TMP/SMX covers a wide antibacterial spectrum, resistance to the agent has developed rapidly among major species of bacteria^5^, ^6^. Antibiotic resistance can lead to treatment failure. Therefore, the appropriate use of TMP/SMX prophylaxis for adult patients with AIN should be carefully determined, which include the optimal dose and schedule.

In conclusion, TMP/SMX prophylaxis may be a useful therapeutic option for managing adult patients with AIN who present with recurrent infections. Further investigations must be conducted to establish the appropriate use of TMP/SMX prophylaxis.

## CONFLICT OF INTEREST

N.A., M.S., and H.M. declare no conflict of interests for this article. H.S. has received consulting fees, lecture fees, and/or honoraria from Mitsubishi Tanabe Pharma, Chugai Pharmaceutical, Astellas Pharma, and Kissei Pharmaceutical and has received research grants from Chugai Pharmaceutical and Astellas Pharma. K.M. has received consulting fees, lecture fees, and/or honoraria from Bristol‐Myers Squibb. The funding source had no role in the design, practice, or analysis of this case report.
